# Genetic Diversity, Structure, and Selective Sweeps in *Spinacia turkestanica* Associated With the Domestication of Cultivated Spinach

**DOI:** 10.3389/fgene.2021.740437

**Published:** 2021-12-08

**Authors:** Sanjaya Gyawali, Gehendra Bhattarai, Ainong Shi, Chris Kik, Lindsey J. du Toit

**Affiliations:** ^1^ Washington State University Mount Vernon Northwestern Washington Research and Extension Center, Mount Vernon, WA, United States; ^2^ Department of Horticulture, University of Arkansas, Fayetteville, AR, United States; ^3^ Centre for Genetic Resources, the Netherlands (CGN), Wageningen University and Research (WUR), Wageningen, Netherlands

**Keywords:** gene flow, genetic diversity, population structure, selective sweeps, spinach, *Spinacia*, SNP

## Abstract

Genotype-by-sequencing (GBS) was used to explore the genetic diversity and structure of *Spinacia turkestanica*, and the selective sweeps involved in domestication of cultivated spinach, *S. oleracea*, from *S. turkestanica*. A total 7,065 single nucleotide polymorphisms (SNPs) generated for 16 *Spinacia oleracea* and 76 *S. turkestanica* accessions placed the *S. oleracea* accessions in one group, *Q*1, and the 76 *S. turkestanica* accessions, which originated from Central Asia, in two distinct groups, *Q*2 and *Q*3. The *Q*2 group shared greater genetic identity with the *S. oleracea* accessions, *Q*1, than the *Q*3 *S. turkestanica* group. Likewise, the *S. oleracea Q*1 group had a smaller *F*st (0.008) with the *Q*2 group than with the *Q*3 group (*F*st = 0.012), and a greater gene flow (Nm = 30.13) with the *Q*2 group than with the *Q*3 group (Nm = 21.83). The *Q*2 accessions originated primarily from Uzbekistan while the *Q*3 accessions originated mostly from Tajikistan. The Zarafshan Mountain Range appears to have served as a physical barrier that largely separated members of the *Q*2 and *Q*3 groups of *S. turkestanica*. Accessions with admixtures of *Q*2 and *Q*3 were collected primarily from lower elevations at the southern end of the Zarafshan Mountain Range in Uzbekistan. Selective sweep regions identified at 32, 49, and 52 Mb on chromosomes 1, 2, and 3, respectively, appear to have played a vital role in the domestication of *S. oleracea* as they are correlated with important domestication traits, including day length sensitivity for bolting (flowering). High XP-CLR scores at the 52 Mb genomic region of chromosome three suggest that a selective sweep at this region was responsible for early differentiation of *S. turkestanica* into two groups in Central Asia.

## Introduction

The genus *Spinacia* consists of cultivated spinach (*Spinacia oleracea* L.) as well as two other species which occur in nature, *S. turkestanica* Iljin and *S. tetrandra* ex M. Bieb (Rubatzky and Yamaguchi, 1997). The three species are diploids (2n = 2x = 12), and all three have an annual life cycle and a dioecious breeding system (Morelock and Correll, 2008). Furthermore, the two wild species are cross-compatible with cultivated spinach. Currently, spinach is grown commercially worldwide. In 2018, 0.9 million ha were cultivated with spinach, producing 26.3 million tons of spinach at a production value of US$18 billion, which accounted for 2% of the global gross annual vegetable production value (FAOSTAT, 2020). *S. tetrandra* is distributed in the Middle East and the Trans Caucasus region (Armenia, Georgia, Iran, Iraq, Kurdistan, and Turkey), and *S. turkestanica* is distributed across Central Asia (Kazakhstan, Tajikistan, Turkmenistan, and Uzbekistan) and South Asia (Afghanistan and Pakistan) (Andersen and Torp, 2011; Ribera et al., 2020; van Treuren et al., 2020). A limited number of *Spinacia* genetic resources is available currently in gene banks around the world, including approximately 2,100 accessions, most of which are *S. oleracea*, with only 89 *S. turkestanica* and 59 *S. tetrandra* accessions (Ribera et al., 2020; van Treuren et al., 2020).

Few genetic diversity studies have been performed on spinach. Diversity analyses were first carried out for spinach accessions using polymerase chain reaction (PCR) assays based on simple sequence repeat (SSR) and target region amplification polymorphism (TRAP) markers to generate genetic fingerprint data (Hu et al., 2007; Khattak et al., 2007; Kuwahara et al., 2014; Göl et al., 2017; Li et al., 2018; Bhattarai et al., 2021). These studies found spinach accessions to be clustered based on their geographic origin, with separation of accessions into two to three major groups. Genotyping-by-sequencing (GBS) has been used in recent years to identify genome-wide single nucleotide polymorphism (SNP) markers (Shi et al., 2017), and transcriptome sequencing has been used to evaluate the genetic diversity and phylogeny of spinach accessions (Xu et al., 2015; Xu et al., 2017). Phylogenetic analysis of cultivated *S. oleracea* accessions and accessions of the wild relatives *S. turkestanica* and *S. tetrandra*, based on transcriptome sequence analysis, revealed that *S. turkestanica* is the progenitor of cultivated *S. oleracea*, with *S. tetrandra* genetically more distantly related to the other two species (Fujito et al., 2015; Xu et al., 2015; Xu et al., 2017). The same conclusion was reached by Ribera et al. (2021) who completed a diversity assessment of wild *Spinacia* species using a limited number of SNP markers (*n* = 56) for 25 *S. turkestanica*, 16 *S. tetrandra*, and 54 *S. oleracea* accessions.

Domestication transforms the traits and genomes of crops (Harlan, 1992; Meyer et al., 2012). During the domestication of wild species, certain traits that are advantageous for humans as sources of food, fiber, and/or other materials are selected, including female inflorescence, enlarged seed and fruit size (Frary et al., 2000; Doebley, 2004); seed dispersal, loss of dormancy, and ripening time (Cockram et al., 2007); flowering time (Xu et al., 2017); and diversification of plant architecture (Clark et al., 2004). During domestication, not only are the phenotypes transformed, but the domestication events also leave genetic signatures, measured as selective sweeps, on both the population structure and genetic diversity of existing populations (Doebley et al., 2006; Abbo et al., 2014). Domestication and the selection of specific agronomic traits reduces genetic diversity across the genome of cultivated species relative to wild species, with a significant reduction in genetic diversity associated with major domestication traits (Doebley, 1989). The genetic diversity and population structure, domestication history, and spread of spinach to current production areas have been examined (Xu et al., 2017; Ribera et al., 2020; Ribera et al., 2021). However, genetic changes in agronomically important traits, and the genetic basis of domestication are not well understood for spinach (Ribera et al., 2020). Xu et al. (2017) reported 93 selective sweeps in the spinach genome that are associated with a number of quantitative trait loci (QTLs), including QTLs for day length sensitivity to induce bolting and flowering, leaf number, stem length, and petiole color associated with domestication of cultivated spinach. A more detailed genetic characterization of available wild and cultivated *Spinacia* accessions may provide further insights into spinach diversity and domestication.

Wild *Spinacia* species can be sources of many commercially important genetic traits (for a review, see: Morelock and Correll, 2008; Simko et al., 2014). For example, the alleles conferring resistance to downy mildew (*Peronospora effusa* f. sp. *spinaciae*) in *S. turkestanica* have been transferred successfully into *S. oleracea*, providing the primary means of controlling this devastating disease (Smith, 1950; Smith and Zahara, 1956; Correll et al., 2011; Ribera et al., 2020; van Treuren et al., 2020). Despite examples of wild species providing valuable genetic traits for cultivated spinach, wild spinach species have not been characterized extensively for economically important traits, and the genetic structure of wild spinach accessions largely has not been explored. Wild relatives of spinach serve as a genetic reservoir for spinach breeding programs and genetic studies, but generally remain untapped. This is due, in part, to limited access to wild spinach accessions since the Convention on Biodiversity (UN, 1992) and the International Treaty on Plant Genetic Resources for Food and Agriculture (ITPGRFA) (FAO, 2009) have become enforced (van Treuren et al., 2020).

In 2008, the Centre for Genetic Resources, the Netherlands (CGN) carried out an expedition to collect seed from populations of *S. turkestanica* at the center of biodiversity for this genus, namely Central Asia (Kik, 2008). After the collecting mission, the seeds were multiplied and added to the CGN gene bank as accessions for public access. The population structure and genetic diversity of *Spinacia* accessions in relation to their geographic origin, the presence of selective sweeps identified using SNP markers, and the relationships of cultivated spinach accessions with accessions of this wild ancestor were investigated in this study to clarify our understanding of the domestication history of spinach. With an extensive SNP data set generated by GBS, this study aimed to address the following questions relating to the origin and domestication of spinach:

1) What is the genetic diversity and group structure of *S. turkestanica* in Central Asia?
2) If a group structure exists, what were the selective sweeps in the *Spinacia* genome that led to differences among these groups?
3) Which *S. turkestanica* selective sweeps were involved in the domestication of spinach?

## Materials and Methods

### Plant Material

A total of 92 accessions of two *Spinacia* spp. was used in this study. Three accessions of *S. turkestanica* originating from Turkmenistan, another *S. turkestanica* accession of unknown origin, and 16 *S. oleracea* accessions were received from the National Plant Germplasm System (NPGS) of the United States Department of Agriculture (USDA). In addition, 72 *S. turkestanica* accessions collected in Uzbekistan and Tajikistan were obtained from the CGN, Wageningen University and Research (WUR). CGN collected the material under the Standard Material Transfer Agreement (SMTA) of the ITPGRFA. Information on each accession, including the country of origin, collection site (latitude, longitude, and altitude), and collection year is provided in Supplementary Table S1. For each accession, one randomly selected plant (representing an accession) was used for the molecular analyses.

Spinach plants were grown in RediEarth propagation mix (Sunshine Horticulture, Agawam, MA) in a greenhouse at the Washington State University (WSU) Mount Vernon Northwestern Washington Research and Extension Center, with the air temperature set at 22–24°C during the day and 18–20°C by night, and supplemental lighting provided for 10 h/day. Plants were fertigated daily with General Purpose Fertilizer 20-20-20 (Plant Marvel, Chicago, IL) injected into the irrigation water at a 1:100 ratio for applying a final nitrogen concentration of 200 ppm at each irrigation. A single leaf was harvested from each plant 35 days after planting, frozen immediately in liquid nitrogen, and stored at −80°C.

### Sequencing and Marker Discovery

Genomic DNA was isolated from the single frozen leaf of each plant using the cetyl trimethylammonium bromide (CTAB) method (Murray and Thompson, 1980; Porebski et al., 1997), after the leaf was ground in liquid nitrogen with a mortar and pestle. The DNA quality was checked on a 1% agarose gel, quantified using a Qubit, and submitted to the University of Wisconsin Madison Biotechnology Center (https://www.biotech.wisc.edu/) for sequencing, where DNA quality and integrity were re-evaluated using the Quant-IT PicoGreen fluorescent dye (Thermo Fisher, Waltham, MA). The GBS method of Elshire et al. (2011) was used to sequence the samples after digesting genomic DNA with the *Ape*KI restriction enzyme, as described by Bhattarai et al. (2020). Digested DNA fragments were ligated with unique barcodes and Illumina adapters, and the samples were pooled in equal proportion to construct GBS libraries, as described by Elshire et al. (2011). Finally, the 96-plex GBS libraries were amplified, purified, and sequenced as 150 bp paired-end reads on an Illumina NovaSeq machine (Illumina, San Diego, CA).

Using the Skewer program (Jiang et al., 2014), the reads were pre-processed to remove sequencing adapters and to filter out low-quality bases for a minimum quality of Q20. Filtered, good quality reads were de-multiplexed and aligned to the spinach reference genome (Xu et al., 2017; http://www.spianchbase.org) using Bowtie two software (Langmead and Salzberg, 2012). The TASSEL GBS v2 pipeline was used to remove barcodes, filter for quality, and call SNPs (Bradbury et al., 2007; Glaubitz et al., 2014). The SNPs were filtered further using VCFtools v0.1.15 (Danecek et al., 2011) to remove indels, remove minor alleles with frequency (MAF) < 0.05, retain only bi-allelic SNPs, achieve a minimum SNP quality score (minQ) < 20 and a minimum genotype read depth (minDP) of 10, remove SNPs missing from >20% of the accessions, and remove plants (accessions) with >20% missing data. Final filtration was used to exclude SNPs with >20% missing data, a minDP of 15, and a MAF <5%. The filtered SNPS were pruned for linkage disequilibrium (LD) in PLINK v1.9 (Chang et al., 2015) using the indep-pairwise 50 5 0.2 option to remove correlated pairs of SNPs. The SNPs were separated into two files, one for each of the *S. oleracea* and *S. turkestanica* species. Both files were checked for common SNPs, and the common SNP was genotyped across all accessions for both species. Final filtered SNP distribution across the six spinach chromosomes was determined using the CMplot package in R. The filtered datasets were then used for diversity, phylogenetic, structure, and selective sweep analyses.

### Population Structure and Clustering

The structure of accessions of the two *Spinacia* spp. was analyzed using the software STRUCTURE 2.3.4, with individual accessions assigned to genetic clusters, hereafter called groups, based on inferred genetic ancestry (Pritchard et al., 2000). Structure implements a Bayesian model-based clustering method which assigns multi-locus accessions to a number of user-defined groups (K), and is based on maximization of linkage equilibrium (LE) within groups but minimization of LE among groups. The structure analysis parameters were set to an admixture model, with K ranging from 1 to 10 using five iterations, a burn in period of 100,000, and a Markov Chain Monte Carlo (MCMC) run length of 100,000. The K was determined by considering various factors suggested by Camus-Kulandaivelu et al. (2006). First, Structure Harvester version 0.6.4 was used to determine K, as described by Evanno et al. (2005) [http://taylor0.biology.ucla.edu/structureHarvester/]. The resulting proportion of membership coefficients (*Q* matrices) for each accession was used to draw a bar plot to visualize clustering of the spinach accessions. True groups were identified as the maximum value of ΔK, based on the rate of change of the natural log probability of the data. *Spinacia* spp. were assigned to individual groups (*Q*) based on an assignment value of 75%. Second, the inferred ancestry assessments obtained from Structure were judged based on prior knowledge of the geographic location of the collection site of each accession of both *Spinacia* spp., provided by the respective gene banks. A total of 7,065 SNPs common to the 76 *S. turkestanica* and 16 *S. oleracea* accessions was used to analyze the population structure of the accessions of these two species. To understand better the group structure and clustering pattern of these accessions, principal component analysis (PCA) was completed using PLINK v1.9 (Chang et al., 2015), and plotted in R. Genetic relationships among the accessions were inferred based on a neighbor-joining phylogenetic analysis in MEGA7 (Kumar et al., 2016) with 200 bootstraps.

The SNPs were used to determine the genetic diversity and genetic structure of the accessions of the two *Spinacia* species. The summary statistics of genetic diversity (GD) and polymorphic information content (PIC) of SNPs were calculated using PowerMarker software V 3.25. (Liu and Muse, 2005). The PIC of SNPs was calculated using the following formula, according to Botstein et al. (1980):

$$PIC=1-\;\overset{n}{\underset{j=1}{\sum }}P_{ij}^{2}-\overset{n=1}{\underset{j=1}{\sum }}\overset{n}{\underset{k=j+1}{\sum }}2P_{ij}^{2}P_{ik}^{2}$$

where, *P*_*ij*_ and *P*_*ik*_ are the frequencies of the *j*th and *k*th alleles, respectively, of bi-allelic SNP marker *i*.

The number of different alleles (Na), number of effective alleles (Ne), Nei’s genetic diversity (*h*) (Nei, 1973; Nei, 1978), unbiased genetic diversity (u*h*), and Shannon’s information index (*I*) were calculated using GenAlEx 6.3 (Peakall and Smouse, 2006). Analysis of molecular variance (AMOVA), group genetic differentiation (*F*st), Nei’s unbiased genetic distance (*D*), and Nei’s unbiased genetic identity (*I*_*d*_) were also estimated using GenAlEx 6.3. Gene flow (Nm) was calculated using the formula Nm = [(1/*F*st)-1]/4, according to Peakall and Smouse (2006). The AMOVA (Excoffier et al., 1992) was used for hierarchical partitioning of genetic variation among the groups, and among individuals within the groups of the two *Spinacia* spp., using *G-*statistics. In total, 999 permutations determined the fixation indices (*F*st) at a level of significance of *p* < 0.001. Nucleotide diversity (π) was calculated using VCFtools v0.1.15 (Danecek et al., 2011) with a 1-kb window size across the genome (window-pi 1,000).

### Linkage Disequilibrium and Selective Sweep Regions in the *Spinacia* Genome

Linkage disequilibrium (LD) was calculated using SNP pairs within a 200 Kb window, and plotted using PopLDdecay3.4.1 (Zhang et al., 2019) for each *S. oleracea* and *S. turkestanica*, and for the *Q*2 and *Q*3 groups of *S. turkestanica* (see details in the Results section). Again, for LD analysis between the *Q*2 and *Q*3 groups of *S. turkestanica*, accessions with <0.75 membership assignment (admixed between *Q*2 and *Q*3) of the group structure were excluded. As a result, of 76 *S. turkestanica* accessions, only 63 (33 from *Q*2 as the query panel, and 30 from *Q*3 as the reference panel) were used. The physical distance of the LD curve intersecting at the critical value of *r* ^2^ = 0.20 was used as LD decay for each species and for groups within *S. turkestanica*. Screening of the spinach genome for selective sweep regions was first performed by comparing allele frequency differentiation between the *S. turkestanica* and *S. oleracea* accessions, following the method described by Xu et al. (2017). Then, since the population structure analysis described above revealed two distinct groups, *Q*2 and *Q*3, among the *S. turkestanica* accessions, screening for selective sweep regions associated with the *Q*2 and *Q*3 groups of *S. turkestanica* was carried out by modelling the likelihood of multi-locus allele frequency differentiation between the two groups using XP-CLR v1.0 (Chen et al., 2010). XP-CLR detects selective sweep regions by modeling the likelihood of multi-locus allele frequency differentiation between two groups. The XP-CLR was run using xpclr v1.1.2 (https://github.com/hardingnj/xpclr) for each pseudochromosome with 50 Kb sliding window, 10 Kb step size, and setting the maximum number of SNPs in each window to 50 (--size 50,000 --step 10,000 --maxsnps 50). The adjacent windows (<10 Kb) with high XP-CLR scores (top 1%) were grouped into a single region representing a single selective sweep region. The candidate genes within the selective sweep regions were also identified. XP-CLR analysis was performed using the 76 *S. turkestanica* accessions as a reference panel and 16 *S. oleracea* accessions as a query panel to identify selective sweeps. Of the 63 *S. turkestanica* accessions that remained after filtering for admixture, 33 accessions in *Q*2 and 30 in *Q*3 were used as the reference panel in the XP-CLR analysis. Similarly, given the evidence (see Results) that the *S. oleracea* accessions (*Q*1) are most closely related to the *Q*2 group of *S. turkestanica*, an analysis of selective sweeps was also computed between the 16 *Q*1 accessions as the query group and 33 *Q*2 accessions as the reference group.

## Results

### Genotyping, SNP Discovery, and SNP Distribution in *S. turkestanica* and *S. oleracea*

Approximately 331.2 million raw reads were generated from the Illumina NovaSeq run for the 92 *Spinacia* accessions. After filtering for sequencing adapters, low-quality bases, and de-multiplexing to extract reads matching the sample barcodes, approximately 300.7 million reads were retained with an average of 3.1 million and a median of 3.1 million reads per accession. Using the TASSEL GBS v2 pipeline, 210,968 SNPs were identified across the six chromosomes. After the SNPs were filtered for indels and a MAF <0.05, 105,328 SNPs were retained. Further filtering to keep only bi-allelic SNPs resulted in retention of 103,704 SNPs.

**FIGURE 1:**
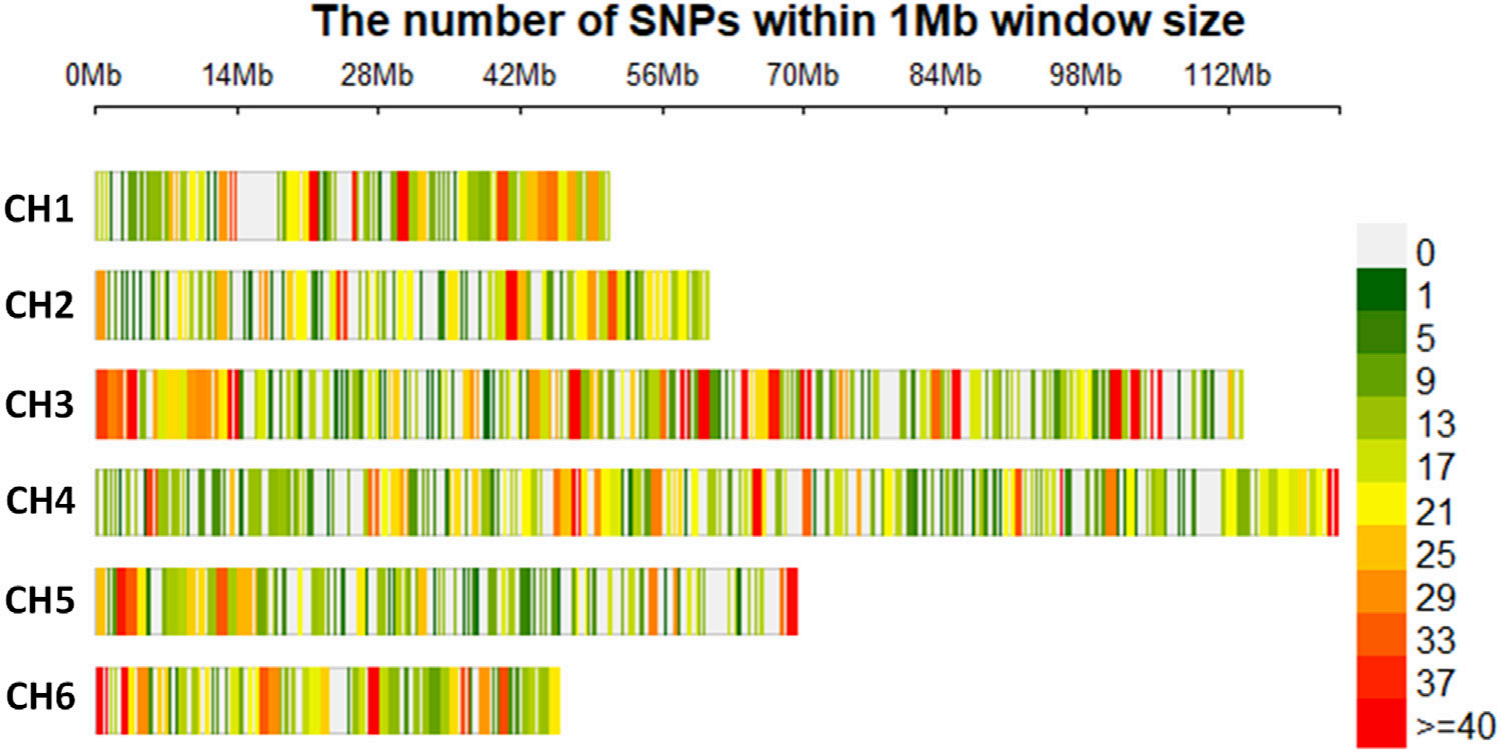
Single nucleotide polymorphisms (SNPs) detected in *Spinacia* genomes after quality filtration, showing the physical density map of 7,065 SNPs common to 16 *S. oleracea* and 76 *S. turkestanica* accessions. The colors refer to the SNP density across the six chromosomes (CH1 to CH6) in the *Spinacia* genome. Numbers above the six chromosome diagrams refer to the distance (in Mb) on the chromosomes of SNPs retained after filtering.

When the SNP dataset was separated into two files, one for each species, with filtering for 20% missing SNP data, 9,456 and 7,629 SNPs were retained for *S. oleracea* and S. *turkestanica*, respectively. Across both species, there were 20.7% missing calls, and the rest of the SNPs were retained for genetic analysis. Among the filtered SNPs, 7,065 SNPs common to all 92 *Spinacia* accessions that met the filtration criteria were retained. The distribution and density of these SNPs across the six chromosomes of *S. turkestanica* and *S. oleracea* are presented in Figure 1, with 832, 839, 1,915, 1,748, 906, and 825 SNPs located on chromosomes 1 through 6, respectively. The SNP density ranged from 0 to >40 SNPs/Mb physical distance. These SNPs were well distributed across all six chromosomes (Figure 1).

### Genetic Diversity, Genetic Differentiation, and Gene Flow in *S. turkestanica* and *S. oleracea*

The genetic diversity of the 7,065 SNPs common to the 16 *S. oleracea* and 76 *S. turkestanica* accessions ranged from 0.10 (40 SNPs) to 0.50 (3,418 SNPs), with an average of 0.35 (Supplementary Figure S1A). The genetic diversity of 79% of these SNPs was >0.3. The PIC of these SNPs ranged from 0.10 (160 SNPs) to 0.40 (3,798 SNPs), with an average of 0.28 for the *S. oleracea* and *S. turkestanica* accessions (Supplementary Figure S1B). The PIC of 75% of these SNPs was >0.3.

The AMOVA of the 76 *S. turkestanica* and 16 *S. oleracea* accessions with 7,065 common SNPs showed 0.5% estimated variance accounted for by the two species, while 99.5% was accounted for by individual accessions within each species (Table 1). The average *F*st and gene flow (Nm) was 44.5 among three groups of the two species. The greatest *F*st, 0.012, was between groups *Q*2 and *Q*3, both of which consisted of accessions of *S. turkestanica*, followed by 0.011 for group *Q*1 of *S. oleracea* and *Q*3 of *S. turkestanica*, and then 0.008 between the *Q*1 and *Q*2 groups (Table 2). The genetic distance, *D*, followed the same pattern, with the largest *D* between groups *Q2* and *Q3*, and the smallest between groups *Q*1 and *Q*2. The genetic identity, *I*_*d*_, was greatest for *Q1* and *Q2* (0.993), followed by *Q1* and *Q3* (0.989), and *Q2* and *Q3* (0.988). Consequently, the highest Nm was between *Q*1 and *Q*2 (30.13), followed by *Q*1 and *Q*3 (21.83), and *Q*2 and *Q*3 (19.77) (Table 2). The mean number of different alleles (Na), number of effective alleles (Ne), diversity index (*h*), unbiased diversity index (u*h*), Shannon’s Information Index (*I*) and nucleotide diversity (π) ranged from 1.988 to 1.997, 1.607 to 1.616, 0.347 to 0.351, 0.355 to 0.357, 0.516 to 0.523, and 0.9223 × 10^−3^ to 0.9371 × 10^−3^ respectively (Table 3). The mean nucleotide diversity (π) of the 92 *Spinacia* accessions was estimated to be 0.8998 × 10^−3^. Overall, of the three groups, *Q*2 was the most diverse (*h* = 0.351, u*h* = 0.356, and *I* = 0.523), followed by *Q*3 (*h* = 0.349, u*h* = 0.355, and *I* = 0.520) and *Q*1 (*h* = 0.347, u*h* = 0.357, and *I* = 0.516). However, the unbiased diversity index (u*h*) of *Q*1, which largely comprised *S. oleracea* accessions, was greater than that of the *Q*2 and *Q*3 groups (Table 3).

**TABLE 1:** Analysis of molecular variance (AMOVA) of the genetic differentiation among and within three subpopulations of *Spinacia* derived from 16 *S. oleracea* and 76 *S. turkestanica* accessions that had 7,065 single nucleotide polymorphisms (SNPs) in common.

Source^a^	df	SS	Est. var.	%^b^	*F*st^c^	Nm^d^	*p* value^e^
Among groups	2	3,483.28	8.17	0.05	0.006	44.5	0.024
Within group	92	151,444.50	1,646.13	99.5			

a Variance partitioned among and within the groups of *S. oleracea* and *S. turkestanica* accessions.

b Percentage of variation among SNPs, contributed among the three groups and within the groups.

c *F*st, Fixation index, a measure of genetic differentiation among populations (Peakall and Smouse, 2006).

d Nm = Number of migrant alleles between subpopulations, Nm=[(1/Fst)−1]/4 (Peakall and Smouse, 2006).

e Probability of obtaining an equal or lower *F*st value, determined with 999 randomizations.

**TABLE 2:** Population differentiation, genetic distance, genetic identity, and gene flow between pairs of three subpopulations of *Spinacia oleracea* and *S. turkestanica* identified using 7,065 single nucleotide polymorphisms (SNPs) common to 16 *S. oleracea* and 76 *S. turkestanica* accessions.

Paired *Spinacia* subpopulation		*F*st^a^	*D* ^b^	*I*_*d*_ ^c^	Nm^d^
*Q*1	*Q*2	0.008	0.007	0.993	30.13
*Q*1	*Q*3	0.011	0.011	0.989	21.83
*Q*2	*Q*3	0.012	0.012	0.988	19.77

a *F*st, Fixation index calculated in GenAlEx 6.5. *F*st provides a measure of the genetic differentiation among populations (Peakall and Smouse, 2006).

b Nei’s unbiased genetic distance (*D*) of the three groups of *Spinacia* in the two species.

c Genetic identity (I_d_) was calculated using GenAIEx 6.5 (Peakall and Smouse, 2006).

d Nm, Number of migrant alleles between species =[(1/Fst)−1]/4 (Peakall and Smouse, 2006).

**TABLE 3:** Mean number of different alleles (Na), number of effective alleles (Ne), diversity index (*h*), unbiased diversity index (u*h*), Shannon’s information index (*I*), and nucleotide diversity (*π*) calculated for 7,065 single nucleotide polymorphisms (SNPs) common to 92 *Spinacia* accessions.

Group (*Spinacia* species)	No. of accessions	Na^a^	Ne^b^	*h* ^c^	u*h* ^d^	*I* ^e^	*π* ^f^
All genotypes^g^	92	1.994	1.611	0.349	0.356	0.520	0.8998 × 10^−3^
*Q*1 (*S. oleracea*)	16	1.988	1.607	0.347	0.357	0.516	0.9371 × 10^−3^
*Q*2 (*S. turkestanica*)	33	1.996	1.616	0.351	0.356	0.523	0.9223 × 10^−3^
*Q*3 (*S. turkestanica*)	30	1.997	1.611	0.349	0.355	0.520	0.9304 × 10^−3^

a Na, number of different alleles.

b Ne, number of effective alleles [ $1/\sum p_{i}^{2}$ ], where *pi* is the frequency of the *i*th allele (Peakall and Smouse, 2006).

c *h*, diversity index $\left[1-\sum p_{i}^{2}\right]$ , where *pi* is the frequency of the *i*th allele (Peakall and Smouse, 2006).

d u*h*, unbiased diversity index [n/(n−1)∗h] , where *h* is the diversity index and *n* is the sample size (Peakall and Smouse, 2006).

e *I*, Shannon’s information index $\left[I=\sum \left(p_{i}^{*}In\left(p_{i}\right)\right)\right]$ , where *p*_*i*_ is the frequency of *i*th allele and *In* is the natural logarithm (Peakall and Smouse, 2006).

f *π*, Nucleotide diversity (π was estimated using VCFtools from 1 Kb windows across the spinach genome).

g All genotypes includes 13 admixed accessions in addition to the *Q*1 (*n* = 16), *Q*2 (*n* = 33), and *Q*3 (*n* = 30) accessions.

**FIGURE 2:**
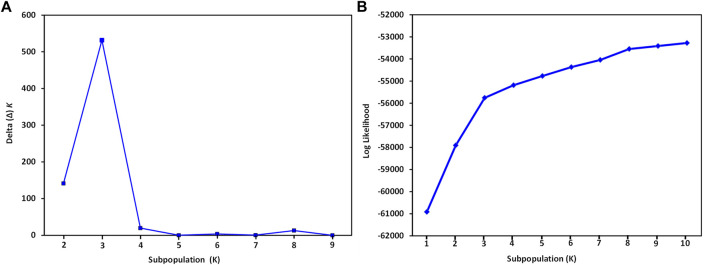
Graphical representation of variance in the number of subpopulations, K, and mean likelihood values generated with the software STRUCTURE for 16 *Spinacia oleracea* and 76 *S. turkestanica* accessions based on 7,065 single nucleotide polymorphisms common to these accessions. **(A)** Delta (Δ) *K* for the different number of spinach subpopulations (K). **(B)** The average log likelihood values of K.

**FIGURE 3:**
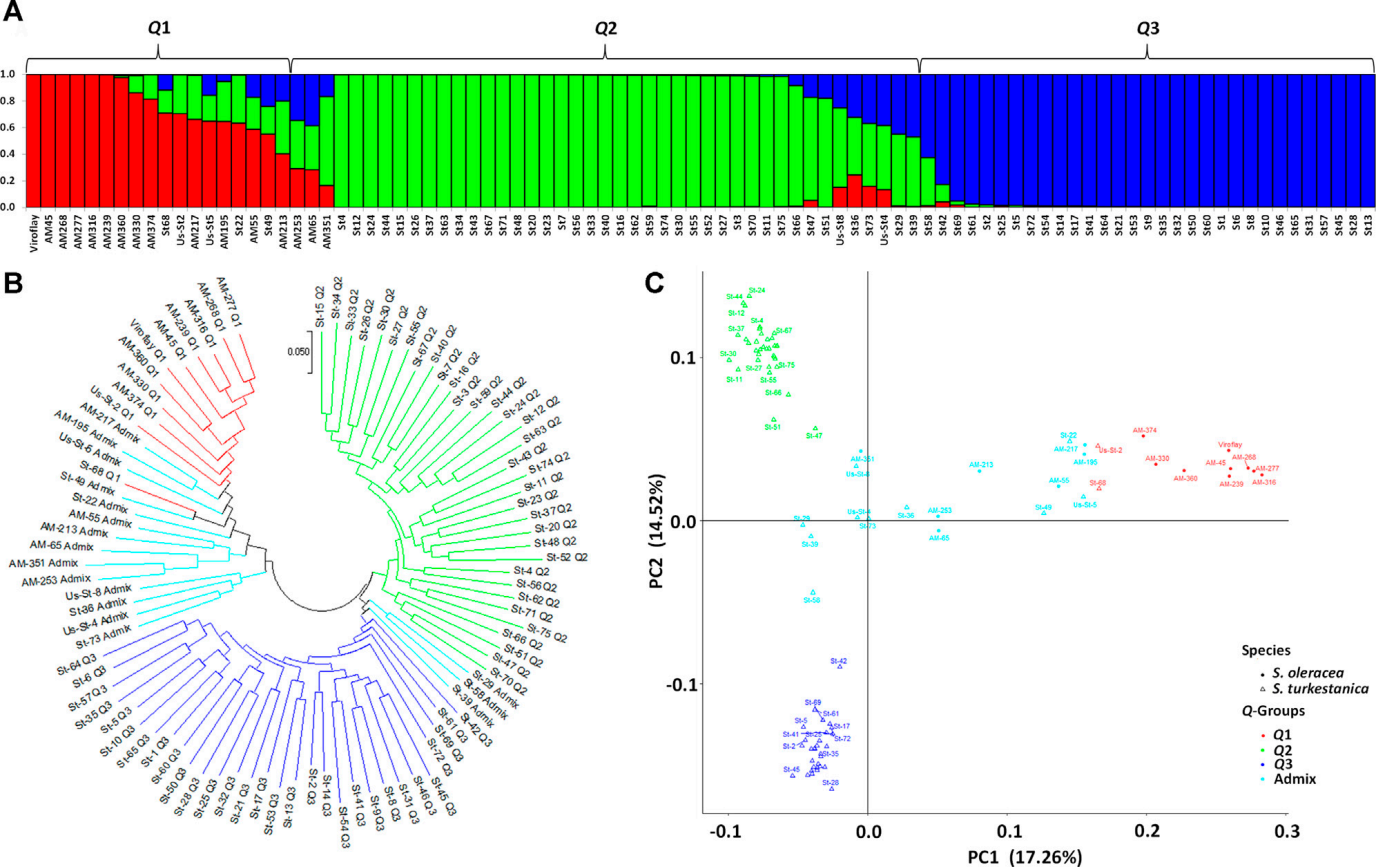
Population structure of 92 accessions of two *Spinacia* spp. based on single nucleotide polymorphisms (SNPs). **(A)** Population structure of 16 *S. oleracea* and 76 *S. turkestanica* accessions based on 7,065 SNPs common to these accessions, with K = 3 groups, *Q*1 (*S. oleracea* and *S. turkestanica*), *Q*2 (*S. turkestanica*), and *Q*3 (*S. turkestanica*), determined using STRUCTURE. **(B)** Neighbor joining tree of the 16 *S. oleracea* and 76 *S. turkestanica* accessions. **(C)** Principal component analysis (PCA) of the 16 *S. oleracea* and 76 *S. turkestanica* accessions based on the SNPs.

The STRUCTURE analysis based on 7,065 SNPs common to the *S. oleracea* and *S. turkestanica* accessions revealed three main groups, *Q*1, *Q*2, and *Q*3 (Figure 2), that comprised 18, 43, and 31 accessions, respectively (Figure 3A). The *Q*1 group largely comprised cultivated spinach, *S. oleracea*, and admixtures of both species, while the *Q*2 and *Q*3 groups comprised *S. turkestanica* accessions only. Multivariate analyses using UPGMA and PCA supported results of the structure analyses, clustering a large majority of the accessions of *Spinacia* into three groups, *Q1* (11 accessions), *Q2* (34 accessions), and *Q3* (30 accessions), with another 17 accessions admixed (Figures 3B,C). The accessions AM45 (United States), AM239 (the Netherlands), AM268 (Macedonia), AM277 (United Kingdom), AM316 (Denmark), AM330 (Nepal), AM360 (United States), and Viroflay (France) had >0.970 inferred membership in the *S. oleracea* group, while another eight *S. oleracea* accessions had varying degrees of admixture with *S. turkestanica* (Figure 3A).

### Geographic Distribution of *S. turkestanica* Accessions Collected in Central Asia

**FIGURE 4:**
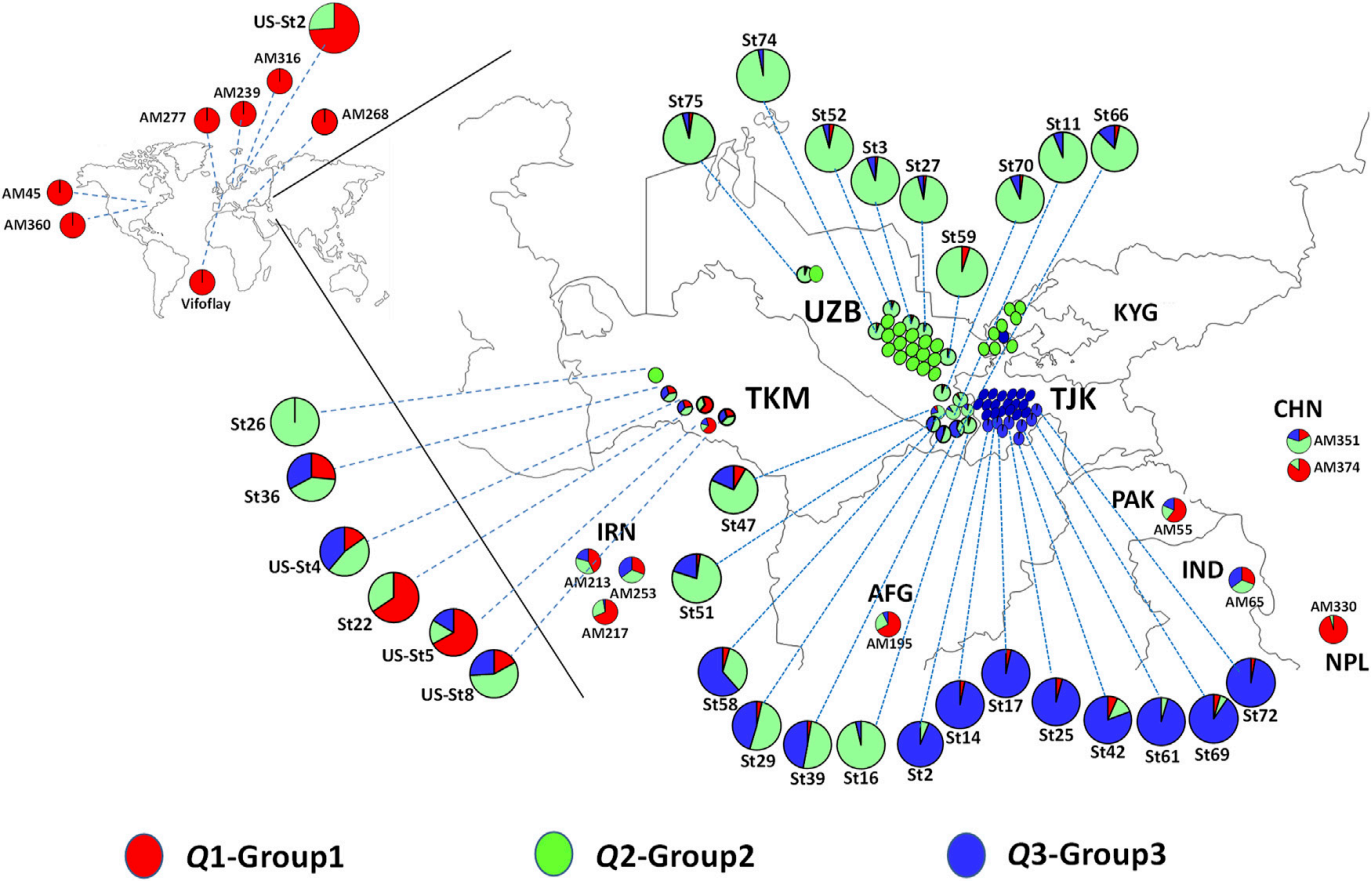
Geographic distribution of accessions of *Spinacia oleracea* and *S. turkestanica* that form three groups based on single nucleotide polymorphisms (SNPs) detected in the plants collected from these locations. Red represents the proportion of membership of a genotype from the *Q*1 group, comprised primarily of *S. oleracea*, and green and blue represent the proportions of membership in two groups, *Q*2 and *Q*3, respectively, of *S. turkestanica*. The admixed accessions are color-coded proportionally to the degree of ancestry from *Q*1, *Q*2, and *Q*3 groups. The *S. turkestanica* accessions consist of mixed ancestry, based on <95% membership in the *Q*2 or *Q*3 subpopulations. AFG, Afghanistan, CHN, China; IND, India; IRN, Iran; KYG, Kyrgyzstan; NPL, Nepal; PAK, Pakistan; TJK, Tajikistan; TMK, Turkmenistan, and UZB, Uzbekistan. AM, Association mapping panel of *S. oleracea* accessions obtained from the United States Department of Agriculture National Plant Germplasm System (USDA NPGS). St = *S. turkestanica* genotype obtained from the Centre for Genetic Resources in the Netherlands (CGN). Us-St = *S. turkestanica* accessions obtained from the USDA NPGS.

The distribution of spinach accessions based on their original geographic location of collection is presented in Figure 4, with detailed information on the two *Spinacia* spp. provided in Supplementary Table S1. The *Q*1 group was dominated by *S. oleracea* landraces (accessions) collected from Afghanistan, China, Iran, India, Nepal, and Pakistan, as well as nine *S. turkestanica* accessions admixed with *S. oleracea* accessions (Figure 4). Of these nine admixed *S. turkestanica* accessions in *Q*1, five (St22, St36, US-St4, US-St5, and US-St8) were collected from Turkmenistan. Interestingly, with few exceptions, group *Q*2 comprised *S. turkestanica* accessions originating from Uzbekistan, while a majority of group *Q*3 comprised *S. turkestanica* accessions originating from Tajikistan (Figure 4). The exceptions included three accessions (St39, St29, and St58) originating from the southeastern part of Uzbekistan, which had varying degrees of admixture of groups *Q*2 and *Q*3.

### Linkage Disequilibrium and Selective Sweep Regions in the *Spinacia* Genome

**FIGURE 5:**
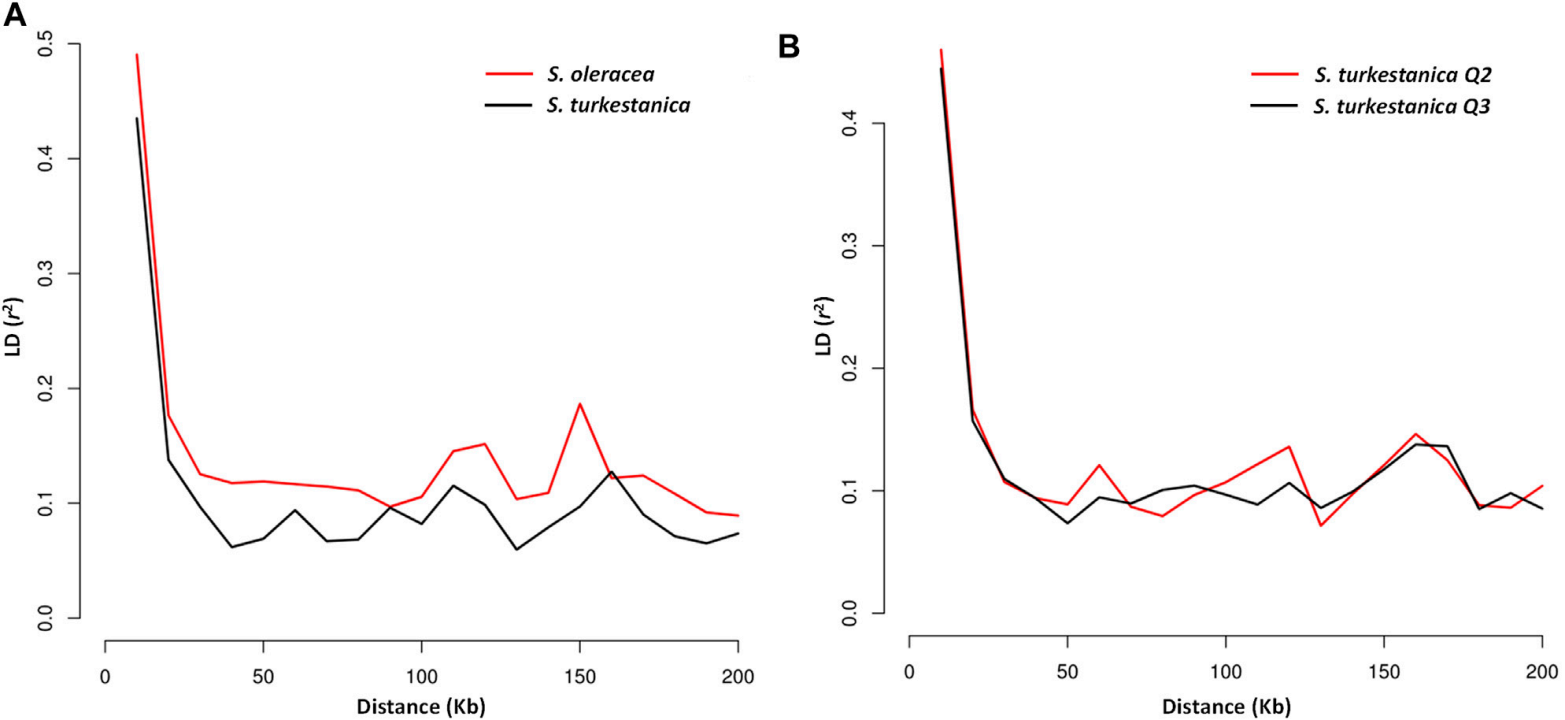
Linkage disequilibrium (LD) decay determined by squared correlations of allele frequency (*r* ^2^) against physical distance (Kb) between single nucleotide polymorphism markers of **(A)** accessions of cultivated spinach, *Spinacia oleracea*, vs. accessions of the wild relative, *S. turkestanica*; and **(B)** group *Q*2 vs. *Q*3 of *S. turkestanica*.

The LD plot showed decreasing LD between markers with the increase in physical distance on the chromosomes (Figure 5). The LD decay plot revealed a rapid rate of LD decay in both *Spinacia* species. The LD decay was around 9 Kb in *S. turkestanica* and around 12 Kb in *S. oleracea* at *r* ^2^ = 0.2 (Figure 5A). The *S. turkestanica* groups *Q*2 and *Q*3 did not show such differences in LD decay rate (Figure 5B). The analysis of selective sweeps of the *S. oleracea* and *S. turkestanica* genomes, determined by XP-CLR analysis, revealed a total of 20 regions, including 3, 2, 9, 1, 3, and two regions in chromosomes 1, 2, 3, 4, 5, and 6, respectively (Table 4; Supplementary Table S2). The highest XP-CLR score was for the 30–32 Mb region of chromosome 1, with 35 SNPs under selection (12 SNPs in chromosome region 1.3 with an XP-CLR score of 24.75009, and 23 SNPs in region 1.2 with an XP-CLR score of 0.00597). Another important selective sweep region was at 98 Mb on chromosome 3, where 23 SNPs are under selection (12 SNPs in region 3.8 with an XP-CLR score of 18.20349, and 11 SNPs in region 3.9 with a score of 11.40893).

**TABLE 4:** Selective sweep regions of 16 *Spinacia oleracea* (*Q*1) accessions and 76 *S. turkestanica* (*Q*2 and *Q*3) accessions, 16 *S. oleracea Q*1 accessions and 33 *Q*2 *S. turkestanica* accessions, and 33 *Q*2 and 30 *Q*3 accessions within *S. turkestanica*, as determined by XP-CLR analyses.

Selective sweep region^a^	Ch	Start (bp)^b^	Stop (bp)^b^	Number of SNPs^c^	Average XP-CLR value^d^	Trait^b^
*S. oleracea Q*1 (*n* = 16 accessions) vs. *S. turkestanica Q*2 + *Q*3 (*n* = 76)						
1.1	1	13767918	13771242	16	2.90262	
1.2	1	30294746	30304212	23	0.00597	
1.3	1	32043580	32067908	12	24.75009	1,3,4
2.1	2	12809167	12824488	15	0.47358	
2.2	2	49380338	49419339	14	0.00005	1,2,3,4,5
3.1	3	42939848	42945383	12	0.00028	
3.2	3	44947407	44947437	13	8.05075	
3.3	3	47715823	47747386	27	2.69194	
3.4	3	49633390	49639104	16	7.17897	
3.5	3	52127066	52127085	10	4.8316	
3.6	3	64263400	64289424	12	7.61429	
3.7	3	64329902	64339621	11	0.08288	
3.8	3	98092802	98118223	12	18.20349	
3.9	3	98111722	98118223	11	11.40893	
4.1	4	37572536	37572740	12	2.3768	
5.1	5	2812557	2856559	15	4.47152	
5.2	5	15307181	15329358	12	3.79875	
5.3	5	68504962	68505353	14	1.01373	
6.1	6	10990114	10995636	11	3.40095	
6.2	6	16607874	16608048	10	6.75217	
*S. oleracea Q*1 (*n* = 16) vs. *S. turkestanica Q*2 (*n* = 33)						
1.3	1	32043580	32067908	12	17.859	1,3,4
2.1	2	12809167	12824488	15	0.606	
3.2	3	44947407	44947437	13	10.170	
3.8	3	98092802	98118223	12	16.506	
3.9	3	98111722	98118223	11	11.017	
3.10	3	11302976	11337711	12	15.832	
4.1	4	37572536	37572740	12	3.798	
4.2	4	91347094	91355343	11	1.031	
5.1	5	2812557	2856559	15	4.862	
5.2	5	15307181	15329358	12	4.054	
6.1	6	10990114	10995630	10	4.680	
6.2	6	16607874	16608048	10	6.311	
*S. turkestanica*: *Q*2 (*n* = 33) vs. *Q*3 (*n* = 30)						
2.3	2	9388527	9388721	11	0.1601	
3.5	3	52127066	52127085	10	19.99774	
3.11	3	1039703	1074601	25	0.00472	
3.12	3	47652733	47654950	11	0.01379	
5.1	5	10484169	10484224	12	5.23574	
5.4	5	67709289	67711500	19	0.92735	
6.3	6	16823978	16824878	11	0.49522	

a Selective sweep region refers to the genomic region (left side of the decimal indicates the chromosome number and right side of the decimal is an ordinal number) that experienced selection during evolution of the spinach genome. Accessions with admixture (see Table 3) were excluded from the analyses, e.g., 13 admixed accessions were excluded from the *Q*2 vs. *Q*3 analysis.

b Start and stop delineate the spinach genomic region (coordinates) of the selective sweep region.

c Number of single nucleotide polymorphisms (SNPs), the total number of SNPs, found in the candidate selective sweep region in the respective spinach genomic region.

d Average XP-CLR, value, the selective sweep value (the higher the XP-CLR, value the lower the allele frequency due to selective sweeps in the genomic region).

e Trait, genetic trait identified by Xu et al. (2017) and Chan-Navarrete et al. (2016): 1, flowering time; 2, bolting; 3, number of leaves; 4, stem length, and 5, petiole color.

Several of the selective sweep regions of *Q*1 vs. *Q*2 accessions were common to the *Q*1 vs. all *S. turkestanica* accessions (*Q*2 and *Q*3), including sweep regions 1.3, 2.1, 3.2, 3.8, 3.9, 4.1, 5.1, 5.2, 6.1 and 6.2 (Table 4). The analysis of selective sweeps of the two groups of *S. turkestanica*, *Q*2 vs. *Q*3, showed a total of seven regions spread over chromosomes 2, 3, 5, and 6 that played important roles in the differentiation of these two groups. Of these selective sweeps, 10 SNPs at the 52 Mb region of chromosome three were common to both *S. oleracea* and *S. turkestanica* and had an XP-CLR score of 19.99774. The next highest XP-CLR score, 5.23574, was for the selective sweep region at 10 Mb on chromosome 5, which encompassed 12 SNPs (Table 4).

## Discussion

The relatively uniform, genome-wide distribution of 7,065 SNP markers identified across all six spinach chromosomes of 16 *S. oleracea* accessions and 76 *S. turkestanica* accessions, and the high genetic diversity and PIC of these SNPs, provided ideal molecular data for genetic analysis of these accessions of cultivated spinach, *S. oleracea*, and accessions of the wild relative, *S. turkestanica*. Botstein et al. (1980) reported that if the PIC of a marker is > 0.5, the marker can be considered highly polymorphic and desirable for genetic studies and crop breeding. The SNPs in this study were bi-allelic, restricting the highest value of PIC to 0.5 when the two alleles of a bi-allelic marker have identical frequencies. However, more than 75% of the SNPs had GD and PIC values > 0.3, so these SNPs are highly desirable for studying the genetic diversity, population structures, and potential breeding strategies for spinach. The nucleotide diversity (π) estimated for cultivated *S. oleracea* accessions (0.9371 × 10^−3^) and the two sub-populations, *Q2* (0.9223 × 10^−3^) and *Q3* (0.9304 × 10^−3^), of wild *S. turkestanica* relatives was similar. The high nucleotide diversity of the 16 *S. oleracea* accessions evaluated in this study might reflect the limited sample size (16 accessions) collected from different geographic regions. The nucleotide diversity estimated using GBS-generated SNPs in this study was slightly greater than estimates in Xu et al. (2017) with transcriptome-derived SNPs that ranged from 0.67 to 0.83 × 10^−3^.

Little information has been published on selective sweeps involved in the evolution of the genus *Spinacia* and domestication of cultivated spinach. This study identified 20 signatures of selective sweeps in the *Spinacia* genome associated with the domestication of *S. oleracea* from *S. turkestanica* (Supplementary Table S2). However, these results should be interpreted with some caution when taking into account that 1 to 40 SNPs per Mb were discovered in this study and a linkage decay (LD) of around 10 Kb can be assumed for cross-fertilizing species like spinach (Lowry et al., 2017). Therefore, it is possible that loci under selection were missed in this study. In this study, selective sweep region 1.3 in chromosome 1, near 32 Mb, had the greatest XP-CLR score, and his region aligns with the QTL for flowering time reported by Chan-Navarrete et al. (2016) and Chitwood et al. (2016). This is also corroborated by Xu et al. (2017) who showed that the selective sweep regions at 1.3 and 2.2 aligned with flowering time, number of leaves, and stem length. Interestingly, the selective sweep region 1.3 found when comparing the *Q*1 *S. oleracea* vs. *Q*2 *S. turkestanica* accessions was not present in the selective sweep analysis of the *Q*2 vs. *Q*3 groups of *S. turkestanica*. The analysis also identified nine selective sweep regions on chromosome 3, with regions 3.8 and 3.9 having high XP-CLR scores of 18.20349 (with 12 SNPs) and 11.40893 (with 11 SNPs), respectively. Further research is warranted to identify the specific domestication traits within these selective sweep regions in *Spinacia*.

Investigation of selective sweep regions of *S. turkestanica* groups *Q*2 and *Q*3 identified seven genomic regions that may be responsible for differentiation of these two groups. None of these selective sweeps regions coincided with domestication traits, although until present little research has been carried out on these traits in spinach. The selective sweep region 3.3 at the 52 Mb region of chromosome three appears to have had an important role in differentiation of the *Q*2 and *Q*3 groups. No genes involved in determining phenotypic traits have yet been associated with this region. A common selective sweep region at the 52 Mb region of chromosome three was also detected between the *S. oleracea Q1* and *S. turkestanica* (*Q2* and *Q3*) groups. This suggests that *S. turkestanica* was differentiated into groups *Q*2 and *Q*3 prior to the domestication of *S. oleracea*. As the *S. oleracea* accessions had greater genetic identity (*I*_*d*_ = 0.993), less genetic distance (*D* = 0.007), and more gene flow (Nm = 30.13) with group *Q*2 accessions compared to group *Q*3 accessions (*I*_*d*_ = 0.989, *D* = 0.011, and Nm = 21.83), it is plausible that most of the *S. oleracea* accessions evolved from the *Q*2 group of *S. turkestanica*.

The orogenic mountain range of Tien Shan stretches through Central Asia, including Kyrgyzstan, Tajikistan, Uzbekistan, and Turkmenistan (Brunet et al., 2017). The Zarafshan mountain range (up to 5,489 m above sea level) within this orogenic belt separates Uzbekistan, where group *Q*2 is concentrated, and Tajikistan, where group *Q*3 accessions were collected. Among the three groups, there was least gene flow (Nm = 19.77) between *Q*2 and *Q*3. This mountain range probably has served as a physical barrier to gene flow between these two groups for thousands of years. Therefore, we hypothesize that the two groups, *Q*2 and *Q*3, of *S. turkestanica* are in an early stage of allopatric speciation. The Zarafshan mountain range of the Tian Shan orogenic belt loses elevation in the southern part, a region where three accessions were found to have mixed genetic constitutions of the *Q*2/*Q*3 groups. This probably points to the occurrence of a hybrid zone between the two groups. Further research is warranted to elaborate on the early speciation of *Q*2 and *Q*3 groups of *S. turkestanica* in Central Asia.

## Conclusions

High throughput GBS was employed to identify SNPs that were then used to explore the genetic diversity, genetic differentiation, and gene flow among accessions of *S. turkestanica* and *S. oleracea*, and to elucidate the origin of cultivated spinach. Three groups were identified among the *S. oleracea* and *S. turkestanica* accessions, with the *S. oleracea* accessions more closely related genetically to the *Q*2 group of *S. turkestanica* accessions than the *Q*3 group of this species. The selective sweep regions identified in the *Spinacia* genome indicated that *S. turkestanica* differentiated into the *Q*2 group (located on the western side of the Zarafshan mountain range) and *Q*3 group (located on the eastern side of the Zarafshan mountain range) first, followed by domestication of cultivated spinach, with *Q*2 accessions of *S. turkestanica* playing a greater role in the domestication of spinach. The selective sweep regions aligned with multiple domestication traits in the 32, 49, and 52 Mb regions of chromosomes 1, 2, and 3, respectively. A hybrid zone between both groups was found at the southern end of the Zarafshan mountain range. The highly polymorphic SNPs identified in this study can be used in future studies, such as genome wide association studies (GWAS) and marker assisted selection for various economically important agronomic traits found in *S. turkestanica*.

## Data Availability

The original contributions presented in the study are available publicly. This data can be found in Figshare at: https://doi.org/10.6084/m9.figshare.15043191.v1.

## Supplementary Material

Supplementary Figure 1 Distribution of the genetic diversity **(A)** and polymorphic information content (PIC) **(B)** for 7,065 single nucleotide polymorphisms (SNPs) common to 16 accessions of *Spinacia oleracea* and 76 accessions of *S. turkestanica*.
